# Reactivity of Aliphatic and Phenolic Hydroxyl Groups in Kraft Lignin towards 4,4′ MDI

**DOI:** 10.3390/molecules26082131

**Published:** 2021-04-07

**Authors:** Leonardo Dalseno Antonino, Júlia Rocha Gouveia, Rogério Ramos de Sousa Júnior, Guilherme Elias Saltarelli Garcia, Luara Carneiro Gobbo, Lara Basílio Tavares, Demetrio Jackson dos Santos

**Affiliations:** 1 Nanoscience and Advanced Materials Graduate Program (PPG-Nano), Federal University of ABC (UFABC), Santo André 09210-580, Brazil; leonardo.dalseno@aluno.ufabc.edu.br (L.D.A.); juliargouveia@gmail.com (J.R.G.); rogerio.sousa@ufabc.edu.br (R.R.d.S.J.); guilherme.elias@aluno.ufabc.edu.br (G.E.S.G.); gobbo.luara@aluno.ufabc.edu.br (L.C.G.); lara.btavares@hotmail.com (L.B.T.); 2 Materials Engineering Graduate Program (PPG-Nano), Federal University of ABC (UFABC), Santo André 09210-580, Brazil

**Keywords:** lignin-based polyurethanes, chemical reactivity, hydroxyl group

## Abstract

Several efforts have been dedicated to the development of lignin-based polyurethanes (PU) in recent years. The low and heterogeneous reactivity of lignin hydroxyl groups towards diisocyanates, arising from their highly complex chemical structure, limits the application of this biopolymer in PU synthesis. Besides the well-known differences in the reactivity of aliphatic and aromatic hydroxyl groups, experimental work in which the reactivity of both types of hydroxyl, especially the aromatic ones present in syringyl (S-unit), guaiacyl (G-unit), and *p*-hydroxyphenyl (H-unit) building units are considered and compared, is still lacking in the literature. In this work, the hydroxyl reactivity of two kraft lignin grades towards 4,4′-diphenylmethane diisocyanate (MDI) was investigated. ^31^P NMR allowed the monitoring of the reactivity of each hydroxyl group in the lignin structure. FTIR spectra revealed the evolution of peaks related to hydroxyl consumption and urethane formation. These results might support new PU developments, including the use of unmodified lignin and the synthesis of MDI-functionalized biopolymers or prepolymers.

## 1. Introduction

Polyurethanes (PUs) are polymers conventionally synthesized through reactions between polyols and diisocyanates. Traditionally, the precursors are derived from petroleum [1]. During the last decades, the development of biobased PUs has attracted significant efforts due to economic and social appeal for sustainable development. Among the renewable precursors, lignin has been standing out, once the resulting material combines the properties of this biopolymer with the polyurethane versatility. Recent technologies for lignin isolation accelerated the scientific and technological interest in lignin. After all, technical grade lignin is readily available on an industrial scale, with low variation in structure and properties. To date, lignin-based polyurethanes were proposed for elastomers [2,3,4], thermoplastics [5,6,7,8], adhesives [7,9,10,11,12], coatings [13,14] and foam formulations [15,16,17,18], among other applications.

**Figure 1:**
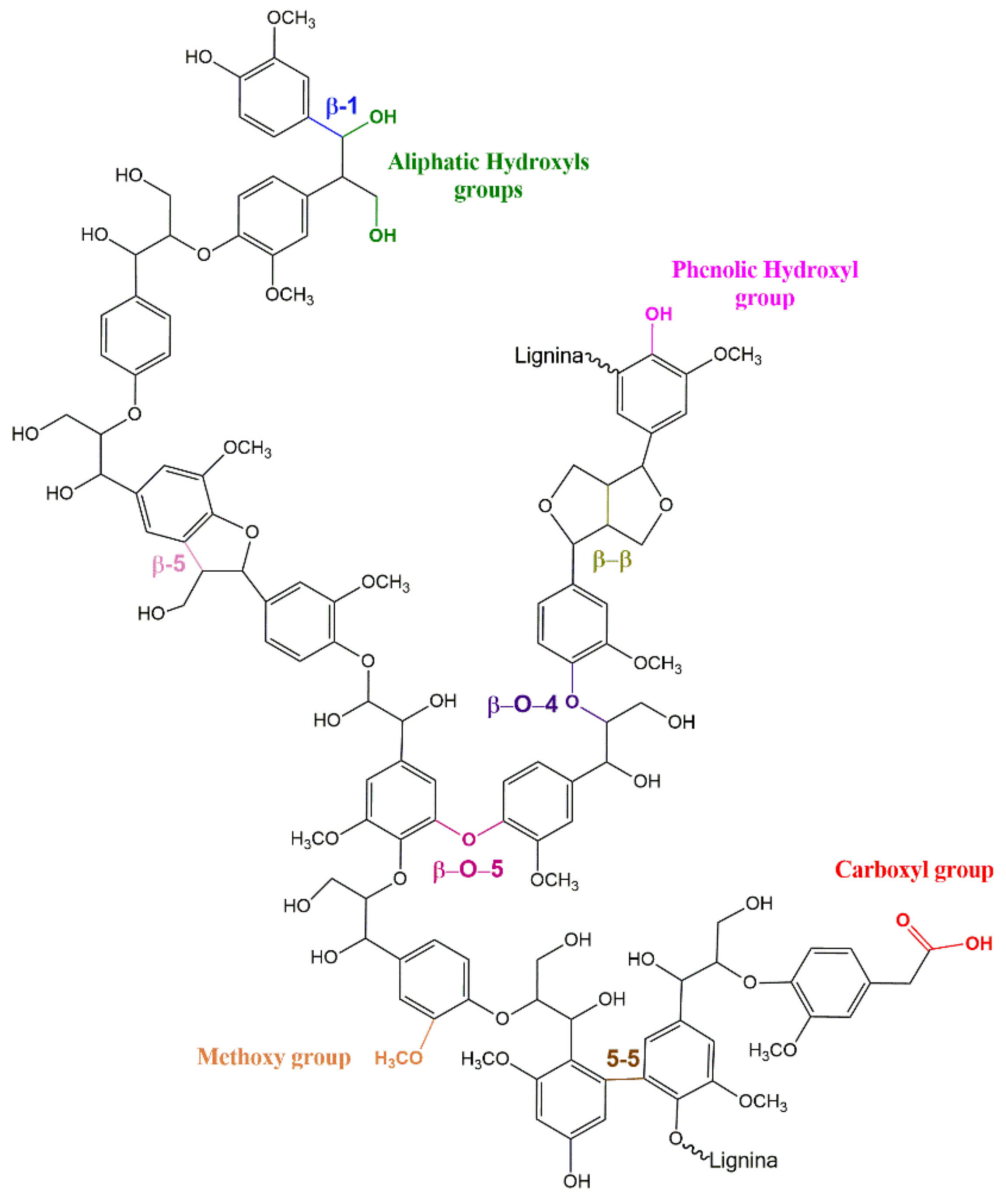
Lignin chemical structure representation. Adapted from Crestini et al. [21].

Lignin is an abundant natural polymer present in plant cells, together with cellulose and hemicellulose. Its amorphous structure is mainly composed by guaiacyl (G-unit), syringyl (S-unit) and *p*-hydroxyphenyl (H-unit) aromatic units, which are linked by carbon−carbon (β–5, 5–5, β–1 and β–β) and carbon−oxygen linkages (β–O–4, α–O–4 and 4–O–5) [19]. In addition, lignin also presents several functional groups: aliphatic and phenolic hydroxyls, carboxyl and methoxyl groups. There are innumerous possible combinations between basic units arising from different synthetic pathways, which make the macromolecule structure complex and heterogeneous [20]. Lignin structure is illustrated in Figure 1. The principal chemical linkages and functional groups are highlighted.

**Figure 2:**
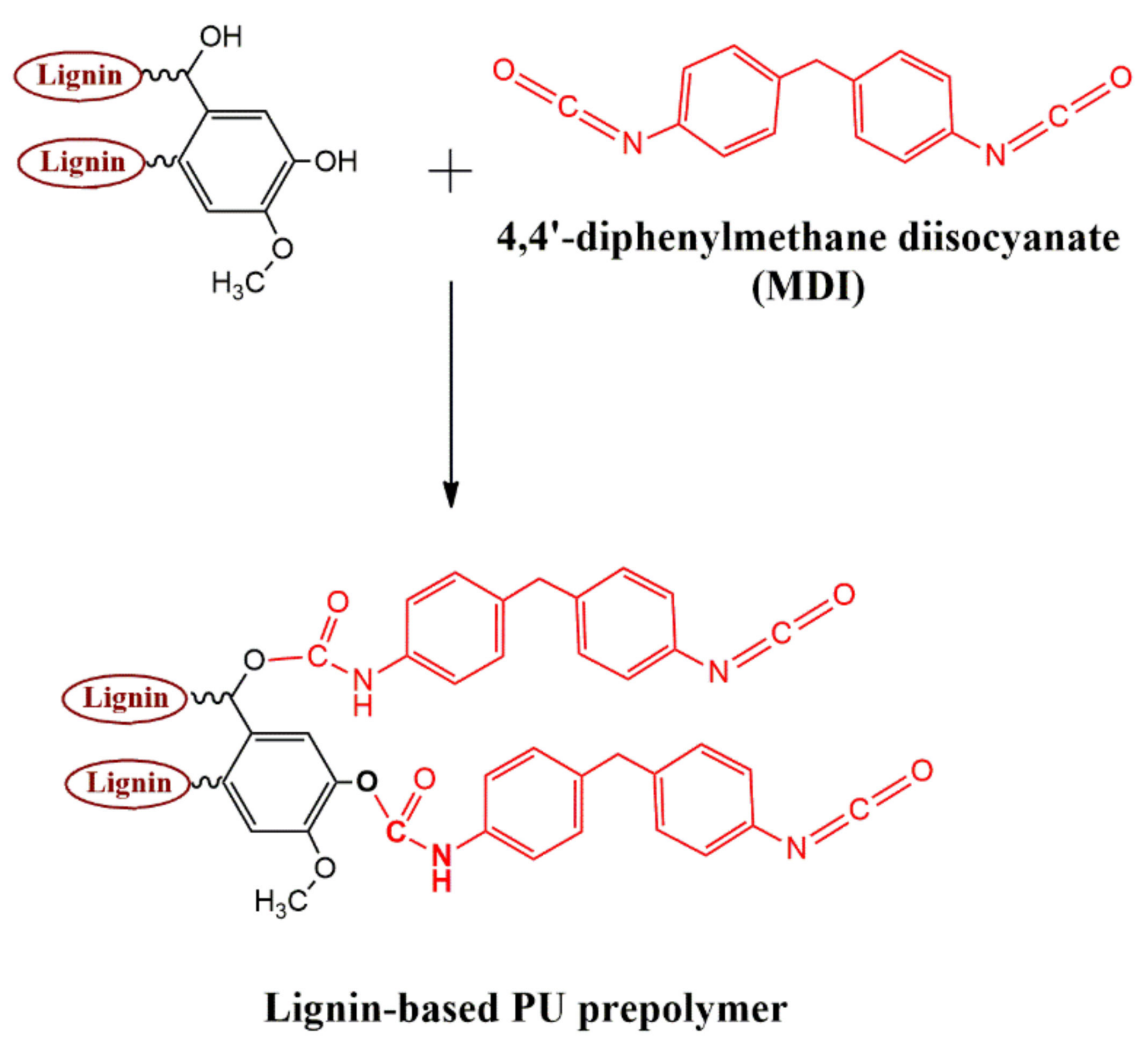
Urethanization reaction of lignin with 4,4′-diphenylmethane diisocyanate (MDI).

**Figure 3:**
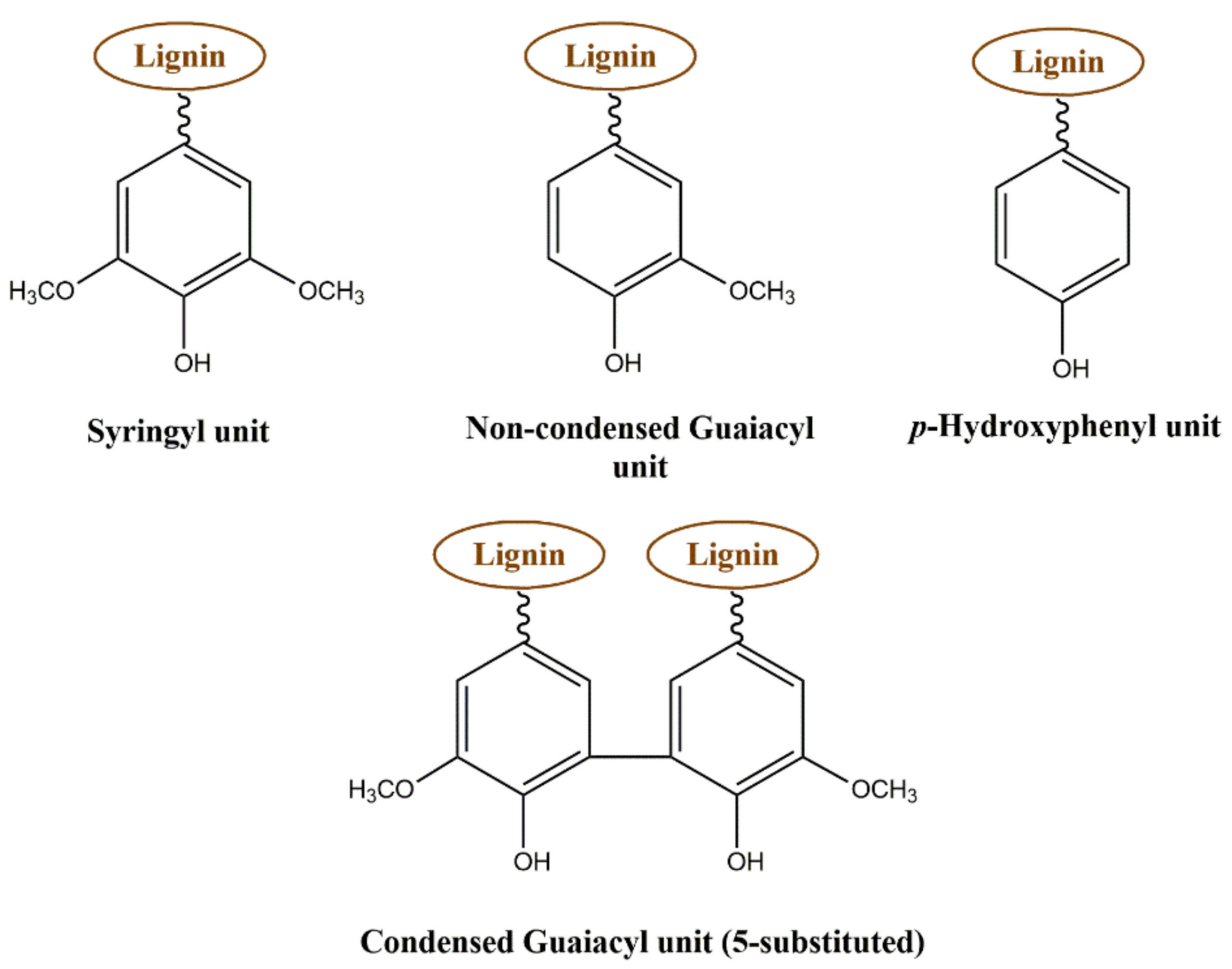
Main types of lignin phenolic OH groups.

Looking specifically at hydroxyl groups, which are the most reactive functional groups of lignin, they promptly react with diisocyanates to form polyurethanes. The reaction mechanism is based on the nucleophilic attack of oxygen from hydroxyl groups on the electrophilic carbon of isocyanate groups [22]. A schematic representation of lignin and 4,4′ MDI is depicted in Figure 2. Hydroxyl groups are abundant in the lignin structure and they have different types randomly allocated, with several levels of reactivity towards diisocyanate. The main hydroxyl groups in lignin are aliphatic (R–OH) and phenolic (Ph–OH). There are also carboxyl ones (R–COOH), but in smaller quantities. Concerning the phenolic hydroxyl groups, they can be divided into four types, according to the unit they are in: syringyl, noncondensed and condensed guaiacyl, and *p*-hydroxyphenyl (Figure 3). Since each Ph–OH type is located in different chemical environments, differences in their reactivity are expected. For this reason, lignin hydroxyl reactivity plays a central role in polyurethane synthesis and might result in phase separation and unsuitable mechanical and thermal properties, if it is neglected during PU formulation and synthesis [5,20].

Considerable scientific efforts have been devoted to control lignin hydroxyl reactivity, which have triggered many modification methods, such as hydroxypropylation [9,15,23], esterification [24] and acetylation [6,7], among other reactions [8]. Indeed, some modification methods successfully converted aromatic hydroxyls into aliphatic ones, minimizing the difference in hydroxyl reactivity levels. More recently, some works reported the partial lignin functionalization with diisocyanate, in which isocyanate-functionalized lignin was developed, with high performance in foams and blend formulations [25,26,27].

Zieglowski et al. [27] prepared different lignin-based isocyanate prepolymers by the modification of Kraft lignin with various diisocyanate compounds. The prepolymers were employed for PU foam synthesis. Additionally, the influence of the diisocyanate molecular structures on the thermomechanical properties and crosslinking reactivity (number of reactive NCO groups during self-crosslinking) was evaluated. However, the reactivity of lignin hydroxyl groups in modification reactions with diisocyanates has not been completely elucidated yet, mainly regarding the selectivity of the isocyanate group for the aliphatic and phenolic hydroxyl groups. Furthermore, the reactivity against isocyanate of different types of phenolic hydroxyls (Figure 3) is scarcely explored in the literature.

Aiming to contribute to the development of lignin-based PUs, this work investigates the reactivity of different lignin–OH groups toward 4.4′ diphenylmethane diisocyanate (MDI). Two technical grade lignins were used, which were obtained from the same industrial process but with differences in pH, molar mass and hydroxyl indexes. The reactivity of each type of –OH was first investigated by nuclear magnetic resonance spectroscopy (^31^P NMR). Fourier transform infrared spectroscopy (FTIR) was performed to support the NMR results. The experimental elucidation conducted could assist in the lignin–isocyanate prepolymers synthesis and, consequently, in the lignin-based PU production, contributing to lignin revalorization.

## 2. Experimental Procedures

### 2.1. Materials

Two types of technical grade Kraft lignin (eucalyptus hardwood) were kindly supplied by Suzano S.A. (Limeira SP, Brazil), with differences in terms of pH, molar mass and –OH content. the biopolymers were designated as Acid Kraft Lignin (Ac_KL, pH = 3.5, 5.61 mmol/g of –OH groups, Mw of 2080 g/mol), and Alkali Kraft Lignin (Alk_KL, pH = 8.1, 5.14 mmol/g of OH groups and Mw of 2830 g/mol). The pH and Mw values were informed by the supplier, whereas the –OH content was obtained from ^31^P NMR quantitative analysis, which is described subsequently. The lignins are isolated from different steps during the extraction process by acidification. According to the supplier, both lignin types exhibit structural repeatability and reproducibility. Tetrahydrofuran (THF), which was used as a solvent, was purchased from Labsynth (Diadema, Brazil). 4,4′-diphenylmethane diisocyanate (MDI) with 33.6% NCO groups (value provided by the supplier), *N*-Hydroxy-5-norbornene-2,3-dicarboximide, *N*,*N*-Dimethylformamide, Pyridine, Chloroform-d, Chromium(III) acetylacetonate and 2-Chloro-4,4,5,5-tetramethyl-1,3,2-dioxaphospholane were used for NMR analysis were purchased from Sigma Aldrich and used as received.

### 2.2. Sample Preparation

The synthesis of isocyanate-functionalized Kraft lignin was based on the procedure previously reported by Chauhan et al. [25]. The lignin samples were previously dried for 24 h in an oven with air circulation at 80 °C to eliminate moisture. In short, 10 g of each type of KL was separately added to 250 mL three-necked round bottom flasks containing 40 mL of THF (inert solvent [25]). Excess of MDI (8:1 ratio between NCO:OH groups) was added to the systems stepwise. The reactions occurred in a closed system under reflux were conducted in air, under stirring at 66 °C (THF’s boiling point [28]), and in an oil bath (glycerol). Temperature variations arising from the exothermic nature of the reactions were neglected. Lignin samples were collected at different time intervals: 15, 30, 60, and 90 min. The modified KL samples were named according to the type of lignin (Ac_KL or Alk_KL) followed by the corresponding reaction time.

### 2.3. ^31^P Nuclear Magnetic Resonance (^31^P NMR)

Quantitative ^31^P NMR spectroscopy (São Carlos, SP, Brazil) was performed to determine the consumption of –OH groups on both types of lignin. The preparation of samples was based on Granata and Argyropoulos work [29], with minor modifications to the procedure: 30 mg of KL was added to 100 μL of endo-N-hydroxy-5-norbornene-2,3-dicarboximide (internal standard) [30]. A solvent mixture of pyridine and deuterated chloroform (1.6:1 *v*/*v*), and relaxation agent chromium acetylacetonate (16.35 mM) were added to the system, which was kept under stirring until complete solubilization of lignin. Finally, 2-chloro-4,4,5,5-tetramethyl-1,3,2-dioxaphospholane (phosphorylating agent) were added to the lignin solution. After solution homogenization, NMR spectra were obtained immediately on a 400 MHz Avance III spectrometer (Bruker BioSpin, Billerica, Massachusetts, United State of America) operating with zgpg30 pulse program (Bruker BioSpin, Billerica, Massachusetts, United State of America) and the following acquisition parameters were used: 25 °C, 512 scans and 25 s delay between pulses. Automatic phase and baseline corrections were performed.

### 2.4. Fourier Transform Infrared Spectroscopy (FTIR)

FTIR-ATR measurements were performed to support the NMR results, based on the consumption of OH groups and appearance of other groups, such as –NH and =C–O, which could be assigned to urethane and urea formation. The analyses were carried out using Spectrum Two (Perkin Elmer, Waltham, Massachusetts, United States of America) equipment in attenuated total reflection (ATR) mode. A total of 32 scans were performed from 4000 to 500 cm^−1^ with a resolution of 4 cm^−1^.

## 3. Results and Discussion

### 3.1. ^31^P NMR

The initial –OH content of both lignin samples are indicated in Table 1.

**Figure 4:**
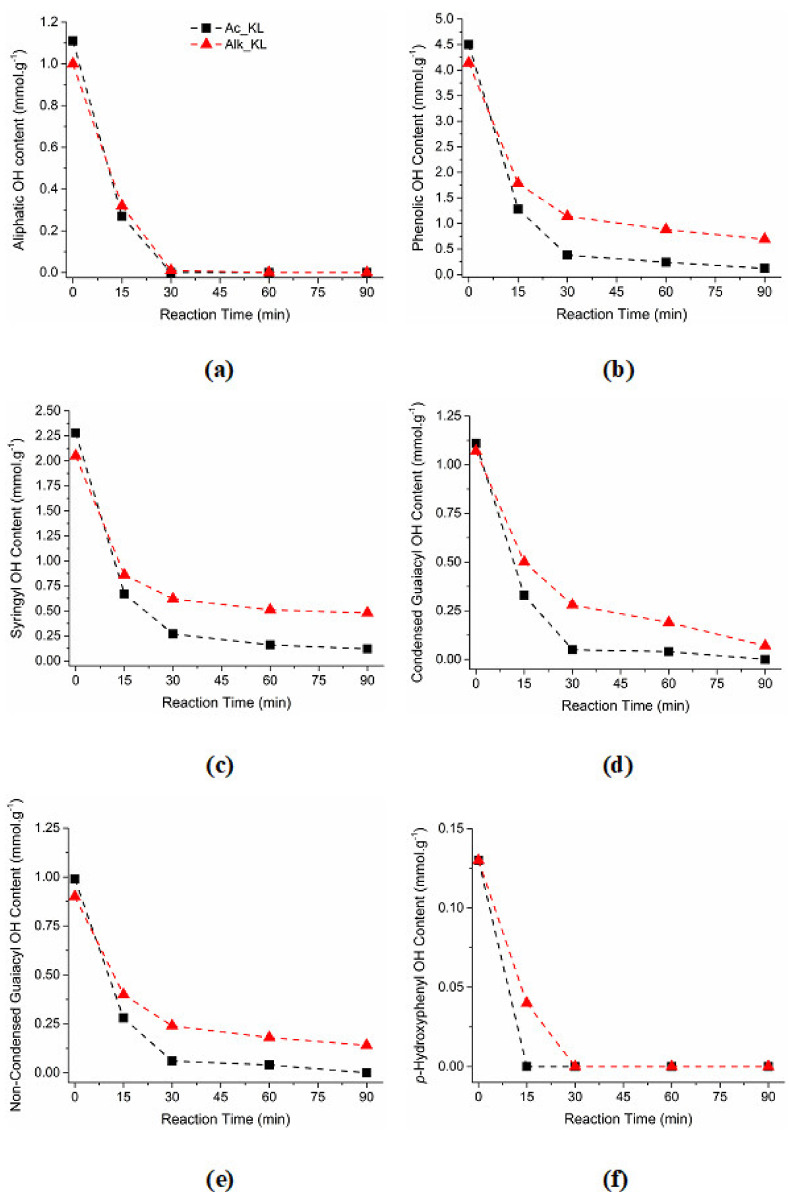
Evolution of hydroxyl group consumption as a function of reaction time during the reaction of Ac_KL and Alk_KL with MDI: (**a**) aliphatic hydroxyl group; (**b**) phenolic hydroxyl group (total); (**c**) syringyl phenolic hydroxyl group; (**d**) condensed guaiacyl phenolic hydroxyl group; (**e**) noncondensed guaiacyl phenolic hydroxyl group; (**f**) *p*-hydroxyphenyl phenolic hydroxyl group.

The lignin hydroxyl groups’ consumption as a function of reaction time was determined by ^31^P NMR quantitative analysis. The data were compiled and are presented in Figure 1. The results indicated heterogeneous reactivity of lignin –OH groups, the major limitation for its use in polymers synthesis, such as polyurethanes [27,31]. A significant difference between aliphatic (Figure 4a) and phenolic hydroxyl groups (Figure 4b) reactivity was observed for both lignin samples. The aliphatic hydroxyl group was totally consumed in 30 min for both lignins, while unreacted phenolic –OH group was verified even after 90 min for both samples. The lower reactivity of phenolic hydroxyl groups towards isocyanates is related to their pronounced acid character compared to its aliphatic counterparts, as well as to steric hindrance effects [22,32]. Usually, the reactivity of aliphatic –OH groups can be up to 1000 times greater in noncatalyzed reactions with isocyanates at room temperature, in comparison with phenolic –OH groups [33]. In addition, the phenolic –OH consumption rate drastically decreased after 30 min. At this time, all the aliphatic –OH was consumed, while the phenolic –OH consumption was 92% and 72% for Ac_KL and Alk_KL, respectively. The lower reaction rate after 30 min might be justified by the steric hindrance, which increased after lignin and MDI reaction.

Differences in reactivity were also observed considering lignin units phenolic hydroxyls, as shown in Figure 4c–f. Firstly, the *p*-hydroxyphenyl –OH group exhibited the highest reactivity (100% consumed after 15 min), whereas the syringyl unit showed the lowest reactivity (94.7% reacted after 90 min). The condensed and noncondensed guaiacyl –OH group showed similar reactivity (both of them were totally consumed after 90 min). For the alkaline lignin type, a similar trend was noted, except for the noncondensed and condensed guaiacyl –OH group, in which the latter presented a higher reactivity (84.4% and 93.5% reacted after 90 min, respectively). This trend is explained by steric hindrance effects acting on lignin phenolic –OH groups [34]. Syringyl, guaiacyl and *p*-hydroxyphenyl phenolic –OH groups have two, one and zero adjacent methoxy groups, respectively (Figure 3). A more pronounced steric hindrance effect is then expected as follows: syringyl > guaiacyl > *p*-hydroxyphenyl –OH groups. Therefore, the reactivity of those groups follows the inverse order, i.e., *p*-hydroxyphenyl > guaiacyl > syringyl –OH groups. The reactivity of condensed guaiacyl –OH group depends to which aromatic ring position other lignin units are linked (only 5 substituted is illustrated in Figure 3) [35]. There are few studies that investigate the reactivity of different types of lignin phenolic –OH groups towards isocyanates using macromolecular models. Most works reported in literature employ models with low molecular weight compounds, as *p*-coumarol, coniferol and sinapol (lignin precursors), and other compounds similar to lignin building blocks, such as guaycol and syringol [22,34,36,37,38].

Comparing the reactivity between Ac_KL and Alk_KL, the first seemed to be more reactive. After 90 min, almost 98% of total –OH groups from Ac_KL were consumed, whereas for Alk_KL this value was down to 87%. Lower molar mass and a higher amount of –OH groups are probably the principal factors that explain the greater reactivity towards MDI of acid lignin type [39,40]. The mobility of chains of low molar mass polymers is greater than high molar mass ones. Then, the –OH groups are more accessible in the Ac_KL than in the Alk_KL, since the MDI diffusion is more favorable in the former. Structural differences also affect the reactivity of lignin–OH groups. For example, syringyl-to-guaiacyl ratio (S/G ratio) and the amount of key linkages, such as β–O–4 linkage, are structural factors that affect lignin reactivity [41]. Therefore, structural changes associated with distinct stages in which acid and alkaline lignins are isolated during the Kraft process can also justify the difference of reactivity between both lignin types.

Herein, the reactivity of –OH groups were evaluated in the context of direct urethanization of lignin with an excess of MDI (NCO:OH = 8). In PU syntheses, in which lower NCO:OH molar ratio and chain extenders (usually polyols with low molecular weight) are employed, the reactivity of these groups is expected to be reduced. Besides lower NCO:OH molar ratio, the expected reduction in lignin–OH group reactivity during PU synthesis is also due to the increase of steric hindrance effect and system viscosity associated with chemical crosslinking [1,32]. The first factor makes –OH groups of lignin less accessible, whereas the second one restricts the diisocyanate and lignin polymer chain diffusion in the reaction medium. For this reason, the reactivity of –OH groups are different when it is evaluated in the context of PU synthesis and direct urethanization, and, thus, should not be compared.

### 3.2. FTIR-ATR

**Figure 5:**
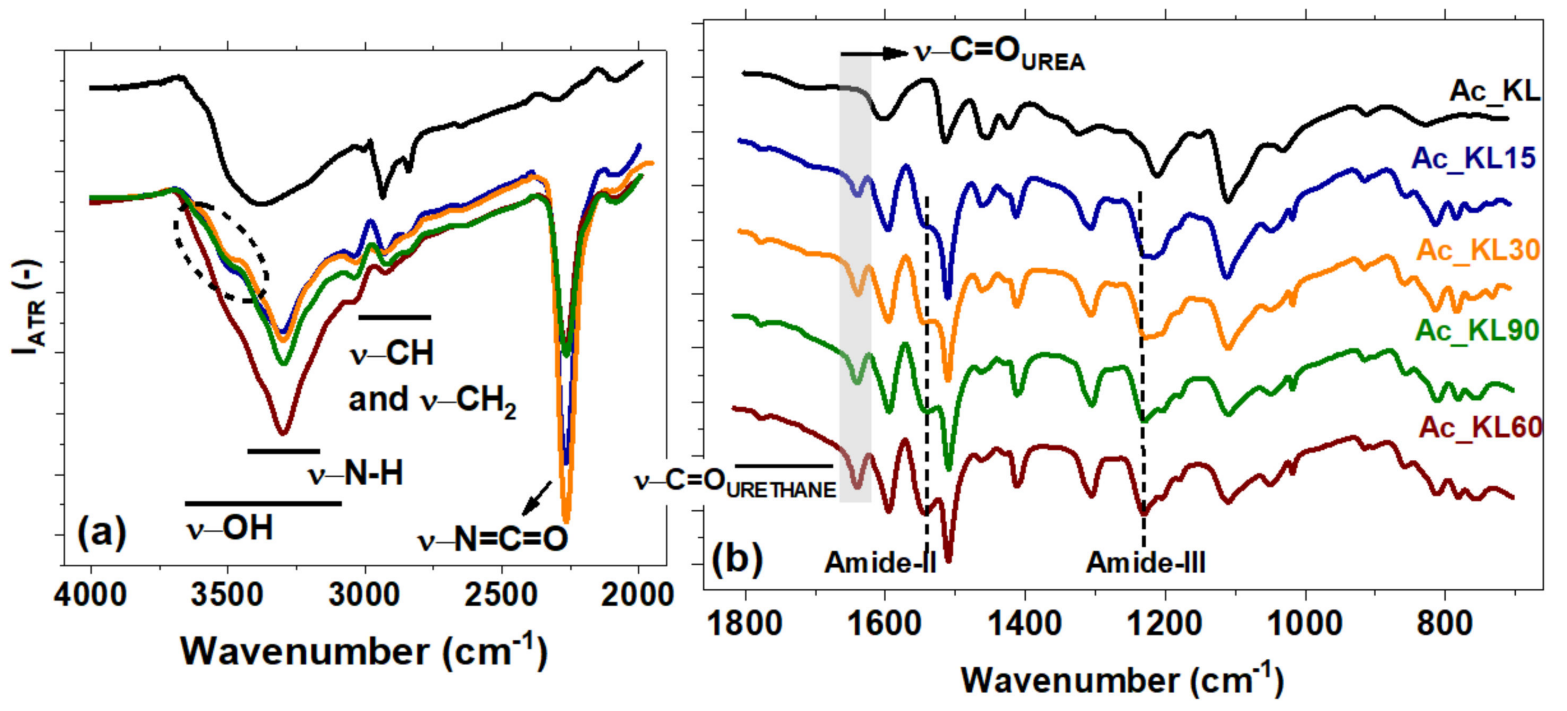
FTIR-ATR spectra of pristine and the derivatized Ac_KL. For clarification, the spectra are shown in two regions (**a**) 4000–1900 cm^−1^ and (**b**) 1800–750 cm^−1^.

**Figure 6:**
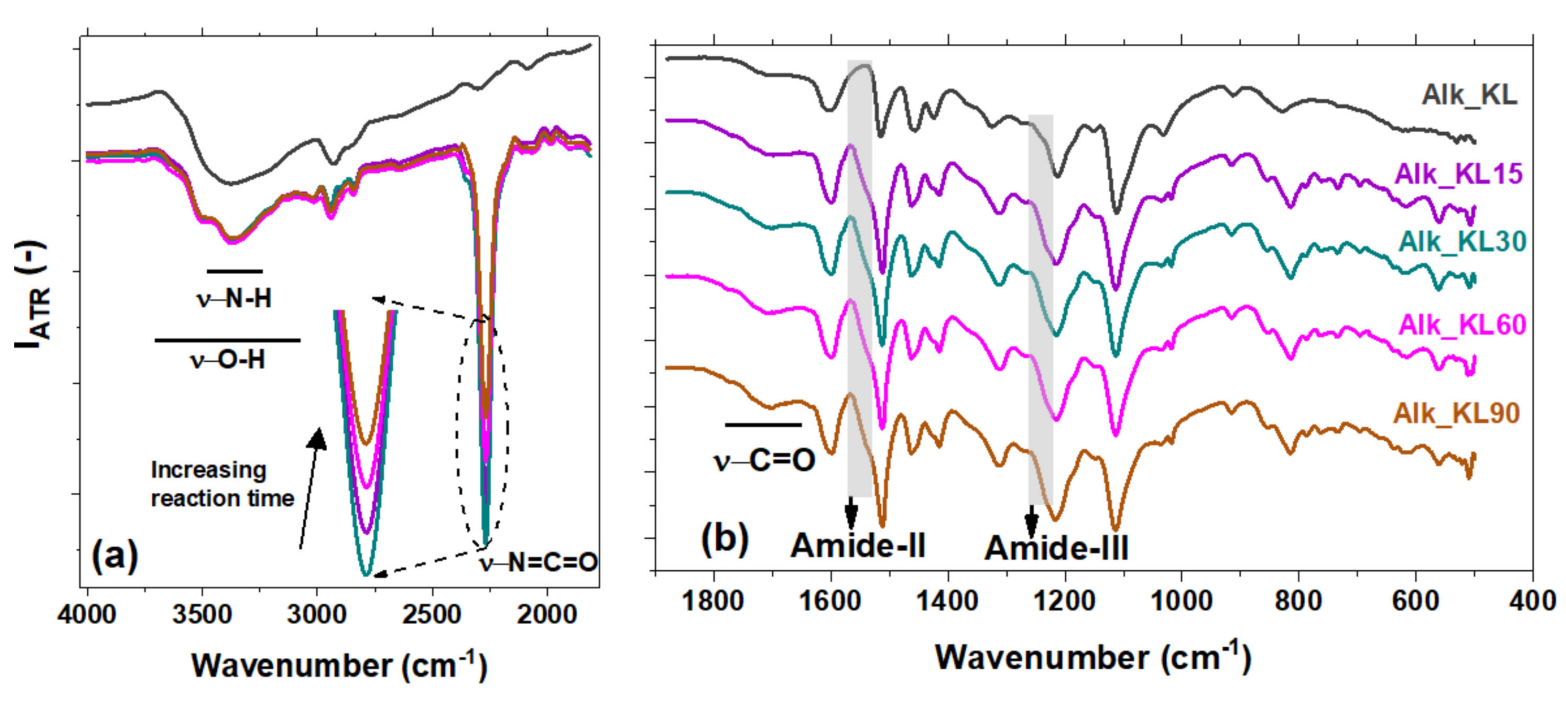
FTIR-ATR spectra of pristine and the derivatized Alk_KL. For clarification, the spectra are shown in two regions (**a**) 4000–1900 cm^−1^ and (**b**) 1800–450 cm^−1^.

The FTIR-ATR spectra of the pristine lignin (Ac_KL and Alk_KL) and their MDI-modified counterpart are shown in Figure 5 and Figure 6, while the main bands and their corresponding assignment are summarized in Table 2.

Because the type of parental lignin affected considerably the reaction with MDI as addressed in the previous section, they will be discussed separately. In the case of the Ac_KL series, several peak changes could be assigned to the urethane formation. In the Ac_KL spectrum, a broad peak attributed to the hydroxyl absorption was found in the region comprised between 3600 cm^−1^ and 3000 cm^−1^. Already at 15 min, this region was marked by the appearance of a sharp peak centered at 3300 cm^−1^, assigned to the amine stretching vibration, which is characteristic of the urethane group [42], whereas a shoulder related to the –OH vibration could still be seen (indicated by the dashed circle in Figure 5a). As the reaction proceeds, the –OH absorption band became less pronounced and disappeared (Ac_KL90 spectra, wine line). The peaks at 2930 cm^−1^ and 2840 cm^−1^ are typically found in the lignin spectra and were assigned to the asymmetric and symmetric vibration of –CH2 and –CH3 groups [43]. The prominent band assigned at 2270 cm^−1^ was attributed to the isocyanate stretching vibration and could be used to estimate kinetic data provided that the spectra are normalized. In this work, we could not find a reference band that could be employed for spectra normalization; therefore, no semi-quantitative analysis could be performed with the FTIR-ATR technique.

The region between 1800 cm^−1^ and 1600 cm^−1^ corresponds to the carbonyl stretching vibration and is of uttermost importance for the investigation of polyurethanes and polyureas. The Ac_KL spectrum showed a small broad peak centered at 1702 cm^−1^, assigned to –C=O band that is present in lignin structure. After reaction with MDI, the carbonyl bands broadened and shifted to higher wavenumbers. As depicted in Figure 5 (black horizontal line), the –C=O peak after MDI-derivatization lay within the urethane carbonyl band. The features in this region suggested the occurrence of a reaction between MDI and lignin–OH that led to the formation of urethane groups [7,12]. This argument is strengthened by the appearance of the so-called amide II and amide III band centered at 1540 cm^−1^ and 1235 cm^−1^, respectively (dashed line) [44]. These bands are related to the combination of the vibration of the –C–N bond, found in the urethane group [45].

**Scheme 1:**
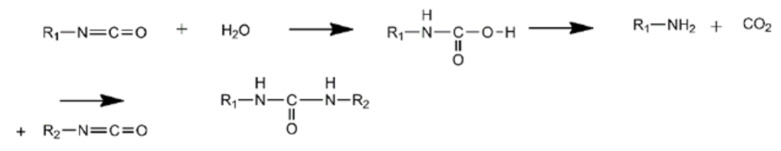
Formation of urea from the reaction of isocyanate and water.

The spectra also revealed that not only urethane groups were formed, but also urea. This observation was supported by the presence of a prominent peak in the –C=O region (1640 cm^−1^) that was attributed to urea. The urea formation is a consequence of isocyanate reaction with water. The side reaction of isocyanate with water is a well-known problem in PU synthesis. As depicted in Scheme 1, the isocyanate and water reaction happens in two stages. First, the unstable carbamic acid is formed, which is decomposed generating CO_2_ and an amine group in the second stage. The amine then is prone to react with the remaining isocyanate, yielding urea groups [46]. The presence of this group in the Ac_KL derivatized spectra was an indication of the presence of humidity in the reaction media. The presence of urea must be taken into account, since it may affect PUs relevant macroscopic properties [47].

Figure 4 shows the same set of plots for the Alk_KL series. Once again, it was not possible to normalize the spectra with respect to each other; however, one may compare the proportional evolution of the peaks within the same curve. While in the Ac_KL derivatized samples the increase in the υ–N–H peak was accompanied by a decrease in the υ–O–H band, in the Alk_KL set the same proportion (i.e., υ–N–H/υ–O–H) was kept throughout the reaction time. This trend suggested that the reaction proceeded in a fast manner in the early stages of the reaction and then reached a plateau. Curiously, the N=C=O vibration peak did not follow the same trend as the υ–N–H/υ–O–H. While the latter overlapped, the intensity of the –N=C=O peak reduced with increasing reaction time, as highlighted by the dashed circle in Figure 6a.

The carbonyl band showed a modest increase with MDI-modification. The same was observed for amide-II and amide-III bands, which appeared only as a shoulder on the peaks from Alk_KL structure (gray rectangles in Figure 6b). Accordingly, no peak assigned to urea could be seen in the spectra. Although no indication of urea formation was identified in the spectra, we do not claim that this group was not formed during the reaction between lignin and MDI. Considering that this reaction was carried out in an open reactor, urea moieties were expected in the lignin-MDI structure, and their absence in the spectra might be a consequence of overlap between lignin/urethane and urea peaks. Collectively, these results suggest an unexpectedly lower reactivity from Alk_KL–OH moieties towards MDI in comparison to Ac_KL [48], corroborating the results obtained in NMR analysis.

## 4. Conclusions

The reactivity of different –OH groups of Kraft lignin toward 4.4′ MDI was investigated. According to ^31^P NMR analysis, aliphatic –OH groups of both lignin types were completely consumed after 15 min, whereas residual aromatic ones were observed after 90 min. ^31^P NMR also revealed a hierarchy reactivity among phenolic hydroxyl groups, in which H units are the most reactive, followed by G and S units. In addition, an unexpectedly high –OH consumption was verified for both lignin types, in all cases higher than 87% of total –OH groups after 90 min reaction. The most reactive lignin presented acidic pH and lower molar mass. FTIR-ATR spectra corroborated the NMR results, confirming a higher consumption of –OH groups for Ac_KL, in which the formation of urea groups was verified. The results confirmed the well-known heterogeneous reactivity of lignin –OHs and suggested that lignin pH influences its reactivity. Although the –OH groups reactivity was nonuniform, a good reactivity between them and isocyanates was verified. Therefore, the unmodified Kraft lignin proved to be feasible for PU prepolymer synthesis. In conclusion, the present work contributes to the development of lignin-based PUs and it might support the development and improvement of modification methods that aim to improve lignin reactivity.

## Figures and Tables

**Table 1:** Hydroxyl content of lignin samples (obtained from ^31^P NMR analysis).

Lignin Sample	Aliphatic OH (mmol/g)	Phenolics OH (mmol/g)					Total OH (mmol/g)
		Syringyl	Condensed Guaiacyl	Uncondensed Guaiacyl	*p*-Hydroxy Phenyl	Total Phenolics	
AC_KL	1.11	2.28	1.11	0.99	0.13	4.50	5.61
Alk_KL	1.00	2.05	1.07	0.9	0.13	4.14	5.14

**Table 2:** Main IR bands assignment of the initial KL and KL-MDI functionalized samples.

Band Positions (cm^−1^)	Assignment
3400	–OH stretching of aromatic and aliphatic
3300	–NH amine stretching
2930	–CH asymmetric stretching vibration of methyl/methylene groups
2840	–CH symmetric stretching of methyl/methylene groups
2270	–N=C=O isocyanate asymmetric stretching
1765–1650	–C=O urethane carbonyl stretching vibration
1702	–C=O lignin carbonyl stretching vibration
1640	–C=O urea carbonyl stretching vibration
1540	Amide II
1235	Amide III

## Data Availability

The data that support the findings of this study are available from the corresponding author, upon reasonable request.

## Sample Availability

Samples of the compounds are available from the authors.
